# Antifungal immune responses: emerging host–pathogen interactions and translational implications

**DOI:** 10.1186/s13073-018-0553-2

**Published:** 2018-05-25

**Authors:** Vinod Kumar, Frank L. van de Veerdonk, Mihai G. Netea

**Affiliations:** 1 University of Groningen, University Medical Center Groningen, Department of Genetics, 9700 RB Groningen, The Netherlands; 2 Department of Internal Medicine and Radboud Center for Infectious Diseases, Radboud University Medical Center, 6525 GA Nijmegen, The Netherlands; 3 Human Genomics Laboratory, Craiova University of Medicine and Pharmacy, 200349 Craiova, Romania

## Abstract

Understanding the complex and highly dynamic interactions between fungi and host cells in a tissue-specific manner is crucial to facilitate the development of new therapeutic approaches to infections. Here, we discuss recent studies that are revealing the mechanisms underlying this context-dependent interplay.

## The mycobiome, fungal infections, and immunity

Fungi are common inhabitants of human barrier surfaces such as the oral cavity, skin, vagina, gut, and lungs. Altered immune status, usually due to treatment with immunosuppressive drugs and sometimes caused by inherited deficiencies in host defense, leads to increased susceptibility to fungal infections. Invasive fungal infections are associated with high mortality rates with an estimated 1.5 million deaths globally each year. Mucosal infections are more prevalent than invasive infections and are a major cause of morbidity. In contrast to bacterial and viral infections, an effective vaccine against fungal infections has not been developed, and currently available antifungal drugs are only partly successful in treating patients with invasive fungal infections. Immunological and genetic studies indicate a crucial role of human immune defects in fungal infections. Therefore, identification of appropriate prophylactic and immunotherapeutic targets has been considered the most promising strategy to overcome morbidity and mortality.

Most invasive fungal infections are caused by species from three genera: *Candida*, *Aspergillus*, and *Cryptococcus*. These fungi can exist in two morphological forms: yeasts (unicellular forms that reproduce asexually by conidia formation) and hyphae (multicellular forms with branching, tubular filaments), which have different cell wall compositions. The hyphal morphotype is usually associated with tissue invasion whereas the conidial form is associated with colonization, which suggests differential host recognition and explains the contrast in virulence.

Fungal pathogens present a variety of pathogen-associated molecular patterns (PAMPs) that may require a unique set of pattern recognition receptors (PRRs) from host cells to recognize and activate distinct downstream immune responses (Table 1). Innate immune cells such as dendritic cells, monocytes, macrophages, and neutrophils are known to express an array of PRRs to recognize fungal infections, to induce protective responses, and to activate adaptive immunity. Roles for different PRRs such as C-type lectin receptors (CLRs), Toll-like receptors (TLRs), and NOD-like receptors (NLRs) in sensing fungal infection and triggering appropriate anti-fungal responses have been established (reviewed in [1]). However, the diverse morphological adaptations (such as conidial and hyphal forms) among fungal pathogens during their interaction with the host immune system, in different tissue compartments and/or different environmental conditions, have hampered efforts to identify therapeutic targets. Recent genetic, genomic, and experimental studies are providing insights into the underlying context-dependent immune mechanisms against fungal infections and the evasion strategies utilized by fungal pathogens, as well as novel host and pathogen targets for the development of potential therapies.

**Table 1:** Human pattern recognition receptors and cell types involved in antifungal immune responses (reviewed in [1])

Fungal pathogen	Routes of infection	Key PAMPs	PRRs	Cell types that express PRRs
*Candida albicans*	Intestine, skin, mucosal surfaces	β-1,3-glucan, O-mannan, N-mannan, chitin, mannose	TLRs (− 2, −4), CLRs (dectin-1, − 2, mincle [7], MR, DC-SIGN, Mcl), NLRs (NLRP3, 4,10), CR3, FcγR, galectin-3, MDA5	Monocytes, macrophages, dendritic cells, neutrophils, mast cells, subset of T cells, B cells, endothelial cells, epithelial cells, gut resident CX3CR1^+^ mononuclear phagocytes [7]
*Aspergillus fumigatus*	Lung	β-1,3-glucan, chitin, galactomannan, DHN-melanin [2]	TLR2, CLRs (dectin-1, − 2, mincle, DC-SIGN), NLRs (NOD1, NLRP3), CR3, PTX3 [3–5], MelLec [2]	Airway epithelial cells, CCR2+ monocytes [9], macrophages, dendritic cells [5], T and B cells, endothelial cells
*Cryptococcus neoformans*	Lung	Mannose, capsular polysaccharide, glucuronoxylomannan	TLRs (−2,-4), CLRs (dectin-2, MR), NLRs (NLRP3)	Macrophages, endothelial cells

*CLR* C-type lectin receptor, *CR3* complement receptor 3, Fcγ receptor, *NLR* NOD-like receptor, *MR* mannose receptor, *MDA5* Melanoma differentiation factor 5, *PAMPs* pathogen-associated molecular patterns, *PRRs* pattern recognition receptors

## Host–pathogen interactions in antifungal immunity

The cell wall of *Aspergillus fumigatus* contains an immunologically active ligand called melanin. In an elegant study, Stappers et al. [2] showed that the lectin receptor MelLec, encoded by the *CLEC1A* gene, is a melanin-sensing CLR, using mouse models and human subjects. This receptor recognizes the naphthalene-diol unit of 1,8-dihydroxynaphthalene (DHN)-melanin present only in conidial spores of *A. fumigatus* and other fungi containing DHN-melanin, but not *Candida albicans* or *Saccharomyces cerevisiae*, which highlights the importance of microbial ligand specificity. MelLec is specifically expressed in mouse endothelial cells, whereas in humans it is ubiquitously expressed in endothelial and myeloid cells. Importantly, a single nucleotide polymorphism (SNP) in the *CLEC1A* gene of human donors that resulted in an amino acid polymorphism (Gly26Ala) in MelLec increased the risk of disseminated *Aspergillus* infections in hematopoietic stem-cell transplant recipients, but this risk was not dependent on recipient SNP genotype. It will be interesting to test whether this polymorphism plays a role in distinct fungal infections in different tissues, which may help to address the question of whether the protection is driven by a pathogen- and/or tissue-specific function of this receptor. Pentraxin 3 (PTX3) is a secreted PRR that is also crucial for host defense against *A. fumigatus* [3]. Recently, polymorphisms in the human *PTX3* gene have also been associated with aspergillosis in patients undergoing hematopoietic stem cell transplantation [4]. Furthermore, downregulation of PTX3 in dendritic cells caused by impaired calcineurin signaling results in higher susceptibility of mice to invasive pulmonary aspergillosis [5]. Administration of PTX3 restores antifungal host responses in humans and mice, but more studies are needed to understand the precise mechanism underlying how PTX3 coordinates the host response against aspergillosis in humans.

Shlezinger et al. [6] unraveled a novel mechanism that underlies how neutrophils in the lung kill *A. fumigatus* conidia, and, conversely, how *A. fumigatus* evades this process. Neutrophils trigger fungal caspase-dependent programmed cell death in the conidia by producing NADPH oxidase, which results in the production of reactive oxygen species and fungal cell death. To evade host-induced programmed cell death *A. fumigatus* expresses the gene *AfBir1*. This gene is homologous to the human *Survivin* gene, which contains a BIR domain that is involved in the suppression of apoptosis by caspase inhibition. These findings highlight the potential for identifying drug targets in the pathogen genome, and suggest that inhibition of *A. fumigatus* AfBir1 could be used to treat invasive aspergillosis, to induce programmed cell death in conidia and improve host survival.

In the human gut, CLRs dectin-1 and dectin-3 are PRRs that have been shown to be important in mediating anti-fungal responses to intestinal fungi (gut mycobiota). Leonardi et al. [7] determined the cell type involved in the regulation of anti-fungal immunity in the intestine. Upon colonization of mouse intestine with *C. albicans*, several fungal PRRs such as dectin-1, dectin-2, and mincle were more highly expressed in gut-resident CX3CR1^+^ mononuclear phagocytes (MNPs) than in dendritic cells. Dendritic cells were previously shown to be important for host defense against fungal infections in the lung. Specific depletion of CX3CR1^+^ MNPs in mice resulted in a reduction in anti-fungal Th17 cells and in IgG antibody responses against intestinal *C. albicans* but not against systemic infection. Thus, CX3CR1^+^ MNPs were specifically involved in innate and adaptive immune responses to intestinal fungi. These findings underscore the importance of tissue-specific cellular functions in fungal infections. Leonardi et al. [7] also investigated the effect of genetic variations in the human *CX3CR1* gene on immunity to fungal infections in patients with inflammatory bowel disease. It is conceivable that because of the immunosuppression treatment strategy used for patients with inflammatory bowel disease, there is an increased risk of intestinal and extra-intestinal fungal infections. A coding polymorphism in *CX3CR1* in patients with Crohn’s disease was associated with impaired ability to produce antibodies against multiple gut fungal species. These findings further identified a role for CX3CR1^+^ MNPs in antifungal immune responses during inflammatory disease. Whether targeting specific cell types such as CX3CR1^+^ MNPs to generate effective antibody responses against pathogenic fungi would be effective in Crohn’s disease patients remains a question for future studies.

Regulation of the antifungal immune response involves coordinated function of many different cell types. Neutrophils and monocytes, which have essential roles in building and modulating the innate immune response, are particularly important in eliminating fungal pathogens, and their roles in regulating interferon (IFN) responses have also been highlighted recently. Using an in vitro infection model and genomics approach, we and others previously showed that the type I interferon (IFN α and β) pathway is strongly activated in response to *C. albicans* infection in human peripheral blood mononuclear cells (which included monocytes and lymphocytes but not neutrophils) [8]. Also, a recent study by Espinosa et al. [9] uncovered another interferon pathway, namely type III IFNs (IFN-λs), as a crucial regulator of antifungal neutrophil responses against *A. fumigatus*. The study also emphasized the importance of context-dependent cellular communication, in which a subset of pulmonary monocytes that express chemokine receptor CCR2 (CCR2+ monocytes) together with neutrophils regulate both type I and type III interferon responses for efficient antifungal responses. In contrast to the antifungal role of gut-resident CX3CR1+ MNPs identified by Leonardi et al. [7], the CCR2+ pulmonary monocytes were important for the antifungal response in the lung [9]. Although the exact cell type that produces IFN-λ is still unknown, observations from survival studies in CCR2-depleted mice upon treatment with IFN-α and IFN-λ cytokines suggest that recombinant cytokine therapies can enhance protective IFN responses and antifungal immunity and could provide potential therapeutic benefits [9].

## Conclusions and future directions

Recent studies have provided important insights into the mechanistic basis for the cellular and organ specificity of host immune responses against fungi, the receptors and pathways involved, and how alterations in these pathways can confer susceptibility to fungal infections in humans. Furthermore, cytokine responses in human peripheral blood mononuclear cells against different fungal and bacterial stimulations have been shown to be strongly dependent on cell type and pathogen type [10]. However, much remains to be discovered about these mechanisms.

Considering the context-dependent regulation of antifungal responses, future studies should focus on systems approaches to comprehensively identify the specific cell types and host and pathogen factors that are involved in orchestrating effective antifungal host responses. Nevertheless, these recent discoveries are stepping-stones towards the design and introduction of effective adjuvant immunotherapy for the treatment of fungal infections.

## Abbreviations

(DHN)-melanin: Naphthalene-diol unit of 1,8-dihydroxynaphthalene (DHN)-melanin
CLR: C-type lectin receptor
MelLec: Melanin-sensing C-type lectin receptor
MNP: Mononuclear phagocyte
NLR: NOD-like receptor
PAMP: Pathogen-associated molecular pattern
PRR: Pattern recognition receptor
TLR: Toll-like receptor
